# MicroRNA-26a-modified adipose-derived stem cells incorporated with a porous hydroxyapatite scaffold improve the repair of bone defects

**DOI:** 10.3892/mmr.2015.3795

**Published:** 2015-05-18

**Authors:** ZHENLIN WANG, DAWEI ZHANG, ZHIQIANG HU, JIWEI CHENG, CHUANMENG ZHUO, XIANCONG FANG, YONGMING XING

**Affiliations:** 1 Department of Orthopaedics, No. 113 Hospital of PLA, Ningbo, Zhejiang 315000, P.R. China; 2 Department of Orthopaedics, Xijing Hospital of PLA, Xi'an, Shaanxi 710032, P.R. China; 3 Department of Otorhinolaryngology, No. 113 Hospital of PLA, Ningbo, Zhejiang 315000, P.R. China

**Keywords:** adipose derived stem cells, miR-26a, osteogenic differentiation, hydroxyapatite scaffold

## Abstract

Tissue-engineered bone substitutes are frequently used to repair bone defects. Adipose-derived stem cells (ASCs) are a promising source of cells for repairing bone tissue, however, insufficient osteogenic potency remains the main obstacle for their application. The present study aimed to enhance the osteogenic potency of ASCs by transfection of microRNA (miR)-26a, a novel osteogenic and angiogenic promoting miRNA. An inverted fluorescence microscope was used to observe transfection efficiency, while a scanning electron microscope was used to detect morphological alterations. Cell proliferation was monitored continuously for 7 days using a Cell Counting kit-8 assay. Osteogenic differentiation was determined by reverse transcription quantitative polymerase chain reaction, alkaline phosphatase (ALP) staining, collagen secretion and extracellular matrix (ECM) mineralization. ASCs were incorporated with a porous hydroxyapatite (HA) scaffold to create a novel tissue-engineered bone substitute and inserted into the critical tibia defect of rats. New bone formation was evaluated by hematoxylin and eosin and Masson's trichrome staining. The results demonstrated that miR-26a was successfully delivered into the cytoplasm, while the morphology and proliferation of ASCs were not significantly altered. Osteogenic-associated genes were markedly upregulated and ALP production, collagen secretion and ECM mineralization were all increased following transfection of miR-26a. Histological evaluation demonstrated that the modified cells accompanied with a porous HA scaffold markedly promoted new bone formation within the defective area. In conclusion, miR-26a transfection significantly improved the osteogenic potency of ASCs suggesting that modified ASCs incorporated with a HA scaffold may be used as a potential bone substitute.

## Introduction

Bone defects are common in the clinic due to increasing incidences of trauma, tumor excision and other deformities (1,2). Despite bone tissue possessing the ability to heal itself, manual intervention is often required to promote repair processes when the defect is larger than the critical size (3). With the development of bone tissue engineering, scaffold-based bone substitutes have resulted in significant progress, among which the hydroxyapatite (HA)-based scaffold is the most frequently used since it best mimics the natural bone composition with an ideal calcium and phosphorus ratio (4). Aside from the scaffold, the cell source is also important in tissue regeneration. Bone marrow-derived mesenchymal stem cells (BMSCs) are the most frequently used, due to their multilineage differentiation capacity (5,6). However, the bone marrow is difficult to obtain in practice, whereas adipose-derived stem cells (ASCs) are easily accessible, which is an advantage for tissue engineering (7). It has already been recognized that ASCs can be used as an alternative to BMSCs for tissue engineering (8,9) and are considered to be a more promising source of stem cells (10). However, the osteogenic capability of ASCs is limited despite being incorporated with an HA scaffold (11) or other scaffolds (12). Consequently, the osteogenic capability of ASCs requires improvement (13).

To date, the recombinant adenovirus vector containing human bone morphogenetic protein 2 (BMP2) has been used to transfect ASCs prior to incorporation with the scaffold (13). However, as the BMP2 gene is exogenous, this may potentially lead to genome modifications. microRNA (miRNA)-based intervention to improve bone anabolic metabolism has been previously investigated and has potential for application in the clinic due to its biosafety and high gene regulatory efficiency (14,15). More specifically, miR-26a has been demonstrated to simultaneously promote osteogenesis and angiogenesis in BMSCs (16). In the present study, it was hypothesized that miR-26a may also modify the function of ASCs incorporated with an HA scaffold to promote new bone generation.

In the present study, primary ASCs were isolated and expanded from rat peritoneal adipose tissue. The miR-26a-modified ASCs were analyzed *in vitro* and the cell loaded HA scaffold was implanted into tibias with a critical sized defect in order to observe its ability to repair bone tissue.

## Materials and methods

### Cell isolation and culture

The animal procedures used in the present study were approved by the Ethics Committee of the School of Medicine, Ningbo University (Ningbo, China). The primary ASCs were isolated and cultured as described previously (17). Specifically, the fresh peritoneal adipose tissue (~2 cm^3^) was isolated under sterilized conditions, washed with phosphate-buffered saline (PBS) and minced, followed by digestion in 0.1% collagenase (type I; Sigma-Alrich, St. Louis, MO, USA) for 40 min in an orbital shaker at 37°C. The digestion was terminated by culture medium containing α-minimum essential medium (HyClone Laboratories, Inc., Logan, UT, USA), 10% fetal bovine serum (HyClone Laboratories, Inc.), 100 U/ml penicillin and 100 mg/ml streptomycin (HyClone Laboratories, Inc.). The mixture was filtered through a cell strainer (100 *µ*m pore size; Falcon, BD Biosciences, Franklin Lakes, NJ, USA) and centrifuged at 120 × g for 5 min. The pellet was then resuspended and maintained in culture medium. Medium was changed twice a week and when cells reached 80% confluence, they were detached with Trypsin (HyClone Laboratories, Inc.) for passage culture. Passages 3–5 were used for further experiments.

### Transfection procedure

The cells were plated at a concentration of 2×10^4^ cells/ml in different cell culture plates (96-well plates for proliferation analysis and 6-well plates for other *in vitro* studies) 24 h prior to transfection. The transfection procedure was performed according to the manufacturer's instructions of the Lipofectamine 2000 transfection reagent (Invitrogen Life Technologies, Carlsbad, CA, USA). Briefly, Lipofectamine 2000, the miR-26a mimic (sense, 5′-UUCAAGUAAUCCAGGAUAGGCU-3′ and antisense, 5′-CCUAUCCUGGAUUACUUGAAUU-3′, denoted as Lipo/miR-26a), the negative control (sense, 5′-UUCUCCGAACGUGUCACGUTT-3′ and antisense, 5′-ACGUGACACGUUCGGAGAATT-3′, denoted as Lipo/NC) or equal volume of RNase-free water (denoted as Lipo) were diluted in Opti-MEM I (Gibco-BRL, Carlsbad, CA, USA) and mixed together. The transfection complex was added directly into the cell culture medium (final miRNA concentration, 50 nM) and incubated for 6 h. Subsequently, the medium was changed to normal culture medium to terminate transfection. The non-treated (NTR) cells were used as a blank control. To observe the transfection process, Cy5-labeled miR-26a was used and the cell membrane was stained with 3,3′-dioctadecyloxacarbocyanine perchlorate (Beyotime Institute of Biotechnology, Haimen, China) at the end of transfection. Images were captured using an inverted fluorescence microscope (Olympus IX70; Olympus, Tokyo, Japan).

### Morphological observation and cell proliferation analysis

To observe the morphology of ASCs, cells were pre-seeded on the cover glass and transfection was performed. Cells were fixed with 2% glutaraldehyde overnight at 4°C 1 day post transfection. Following dehydration in a series of graded ethanol, the specimens were sputter coated with platinum and observed by scanning electron microscopy (SEM; S-4800; Hitachi, Tokyo, Japan). For proliferation analysis, the ASCs were seeded into a 96-well plate and transfected. The cell proliferation was represented by the cell viability, which was determined using a Cell Counting kit-8 (CCK-8) kit (Beyotime Institute of Biotechnology) continuously for 7 days. Briefly, the medium was removed and replaced with 100 *µ*l reaction solution (CCK-8: medium=1:10). Following incubation for 3 h, the supernatant was transferred into a new 96-well plate and the absorbance was read at 465 nm using a microplate reader (Bio-Tek Instruments, Inc., Winooski, VT, USA).

### Reverse transcription quantitative-polymerase chain reaction (RT-qPCR) analysis

Following seeding and transfection, the cells were allowed to proliferate for 3 days in culture medium and then the medium was replaced with osteogenic medium containing 10 mM β-glycerophosphate (Sigma-Aldrich), 50 mg/ml ascorbic acid (Sigma-Aldrich) and 10^−7^ M dexamethasone (MP Biomedicals, Santa Ana, CA, USA) for a further 3 and 7 days. Total RNA was then isolated using TRIzol reagent (Invitrogen Life Technologies) and reverse transcribed to complementary DNA (cDNA) using a PrimeScript™ RT reagent kit (Takara Bio, Inc., Shiga, Japan). Normalized cDNA was used for amplification with an SYBR Premix Ex™ Taq II RT-PCR kit (Takara Bio, Inc.) in an Applied Biosystems 7500 Real-Time PCR System (Applied Biosystems, Foster City, CA, USA). RT-qPCR was performed to measure the osteogenic-associated genes, including alkaline phosphatase (ALP), collagen I (COL1), osteocalcin (OCN) and bone morphogenetic protein 2 (BMP2). Glyceraldehyde 3-phosphate dehydrogenase was used as an endogenous reference. The primers are listed in Table I.

### ALP quantification

The cells were cultured and induced as described above. ALP quantification was performed 7 days post induction based on measuring the rate of p-nitrophenol formation from p-nitrophenyl phosphate (Sigma-Aldrich). Briefly, the cells were rinsed with Tris-buffered saline and fixed in 3.7% formaldehyde in 90% ethanol for 30 sec at room temperature. Following removal of fixation, the reaction substrate solution (1 mg/ml P-nitrophenyl phosphate in 50 mM NaHCO_3_, at pH 9.6 with 1 mM MgCl_2_) was added and incubated at 37°C for 20 min. NaOH was added to terminate the reaction and the absorbance was measured at 405 nm to represent the relative ALP expression. ALP activity was expressed as nanomoles of p-nitrophenyl produced per minute per microgram of protein.

### Osteogenic staining assay

The cells were treated and induced as described for the RT-qPCR analysis. After 14 days of induction, the medium was removed and rinsed with PBS followed by fixation in 4% paraformaldehyde for 15 min. ALP staining was performed with a BCIP/NBT Alkaline Phosphatase Color Development kit (Beyotime Institute of Biotechnology) for 30 min. Collagen secretion and extracellular matrix (ECM) mineralization was stained with Sirius Red (0.1 wt% in saturated picric acid) and Alizarin Red (40 mM, pH 4.2), respectively. Unbound dye was removed with 0.1 M acetic acid or distilled water and then images were captured using an inverted optical microscope (IX70; Olympus).

### ASC-loaded HA scaffold implantation

The rat tibial defect model was produced according to a previous study (18). A total of 20 male Sprague Dawley rats (6–8 weeks old) were purchased from the School of Medicine, Ningbo University and maintained in clean conditions with a normal diet. Animals were anesthetized with sodium pentobarbital (Sigma-Aldrich). A 3.5 mm diameter cortical defect was created and implanted with different pretreated ASC-loaded HA scaffolds (Jiangsu Yenssen Biotech Co., Ltd., Jiangyin, China). Animals were sacrificed 12 weeks post implantation and the tibia was isolated and fixed immediately in 4% formaldehyde.

### SEM

The specimen was dehydrated with ethanol and freeze-dried. The dried sample was mounted by a double side carbon tape, sputter coated and observed by SEM (S4800; Hitachi).

### Histological evaluation

The tibia specimen was decalcified in 20% EDTA (pH 7.2–7.4) over 2 weeks. Decalcified tissue samples were embedded in paraffin and sliced into 3 *µ*m thick sections. Histological evaluation was performed with routine hematoxylin and eosin (H&E) and Masson's trichrome staining. Images were captured using an optical microscope (Olympus) following being sealed in neutral balsam.

### Statistical analysis

Three independent biological experiments were repeated and the quantitative data are presented as the mean ± standard deviation. One-way analysis of variance accompanied with Student-Newman-Keuls test were performed to compare the means. P<0.05 was considered to indicate a statistically significant difference.

## Results

### Transfection, morphology and proliferation

**Figure 1:**
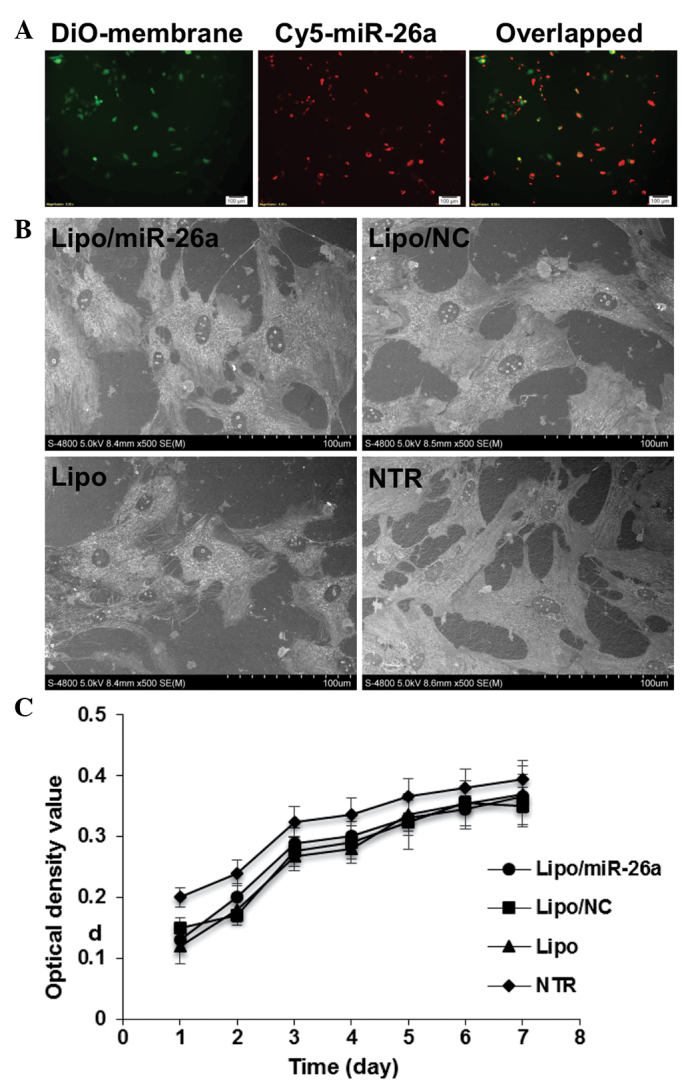
(A) Observation of transfection using a fluorescence microscope 6 h after transfection (magnification, ×40). (B) Observation of cell morphology using a scanning electron microscope after 1 day of transfection (magnification, ×500). (C) Proliferation curve measured by Cell Counting kit-8 continuously for 7 days. NTR, non-treated; Lipo, Lipofectamine 2000; NC, negative control; DiO, 3,3′-dioctadecyloxacarbocyanine perchlorate; miR, microRNA.

The cells were observed under an inverted fluorescence microscope 1 day post transfection. Cy5-labeled miR-26a was able to collocate with cells, which confirmed successful transfection (Fig. 1A). The cells spread well with abundant pseudopodia and protuberances in all groups and no particular morphological alterations were observed following miR-26a transfection (Fig. 1B). The proliferation curve was obtained by optical density measurement following incubation with CCK-8. Cell viability increased in each group and no statistical differences were observed (Fig. 1C). A partial suppression was observed in the lipofectamine only group and the NTR cells had the highest viability at each day, suggesting that the partial suppression in viability in the transfection groups may be due to lipofectamine cytotoxicity (Fig. 1C).

### RT-qPCR and ALP quantification

**Figure 2:**
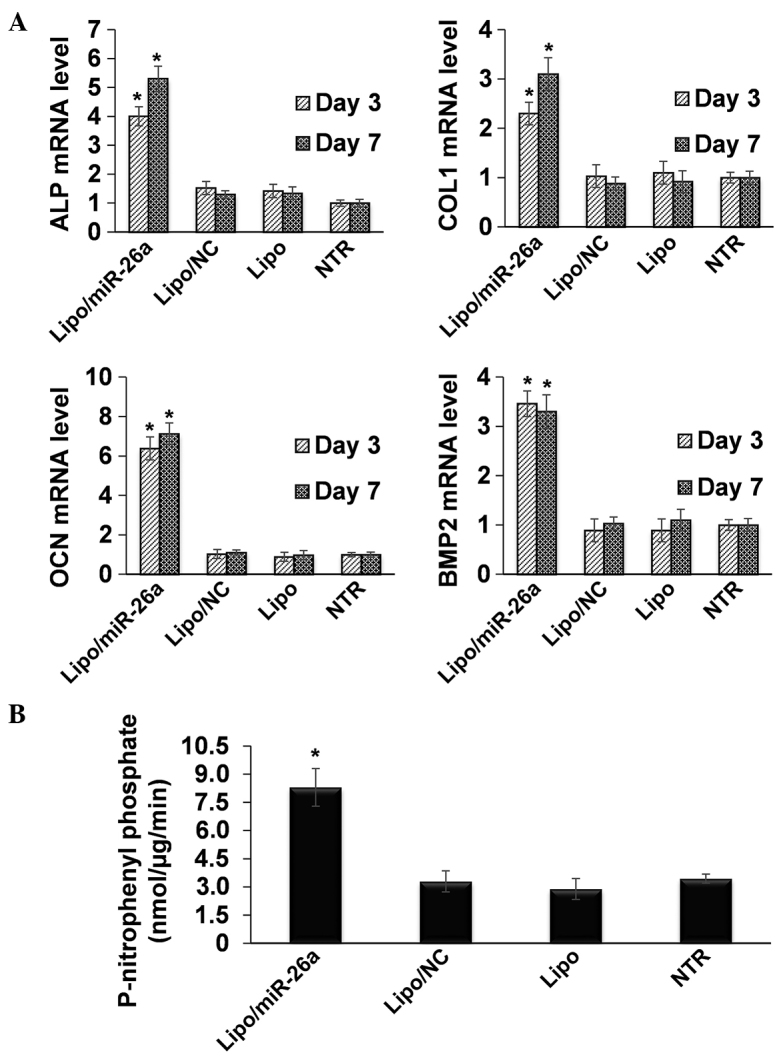
(A) Reverse transcription-quantitative polymerase chain reaction of osteogenic-associated genes and (B) ALP quantification. ^*^P<0.05 vs. Lipo/NC, Lipo and NTR. NC, negative control; NTR, non-treated; ALP, alkaline phosphatase; COL1, collagen I; OCN, osteocalcin; BMP2, bone morphogenetic protein 2; miR, microRNA; Lipo, Lipofectamine 2000.

In order to analyze the promotion of osteogenic differentiation following transfection of miR-26a, RT-qPCR was performed to measure the expression of osteogenic-associated genes. As expected, all the genes measured in the present study were markedly upregulated following transfection of miR-26a (Fig. 2A). Specifically, ALP increased ~4-fold on day 3 and >5-fold on day 7 (Fig. 2A). COL1 increased >2-fold on day 3 and ~3-fold on day 7 (Fig. 2A). The expression of OCN increased by >6-fold on day 3 and >7-fold on day 7, while the mRNA expression of BMP2 increased >3-fold at the two time points (Fig. 2A). Quantification of ALP protein expression was further performed based on the enzyme catalytic reaction. The NTR sample expressed ALP ~3 nmol/*µ*g/min after 7 days of induction in osteogenic medium, which was significantly increased to ~8 nmol/*µ*g/min following miR-26a transfection (Fig. 2B).

### Osteogenic staining analysis

**Figure 3:**
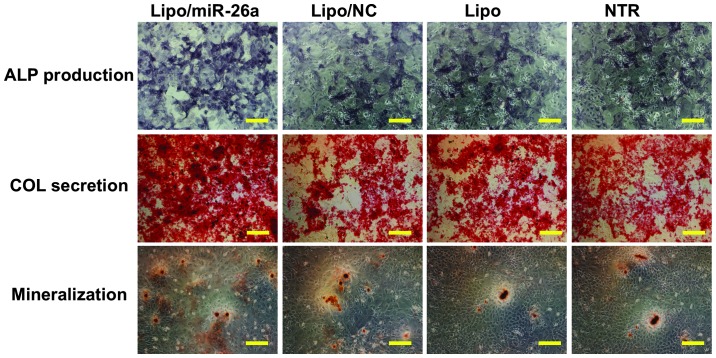
ALP production, COL secretion and extracellular matrix mineralization by BCIP/NBT, Sirius Red and Alizarin Red staining, respectively. Scale bar=100 *µ*m. NC, negative control; NTR, non-treated; ALP, alkaline phosphatase; COL, collagen; miR, microRNA; Lipo, Lipofectamine 2000.

ALP production, collagen secretion and ECM mineralization were analyzed. In accordance with the RT-qPCR results, ALP production and collagen secretion was abundantly detected in the miR-26a transfection group (Fig. 3). In addition, the calcium nodules in the miR-26a group were also more prominent compared with the control groups (Fig. 3).

### SEM observation and histological evaluation

**Figure 4:**
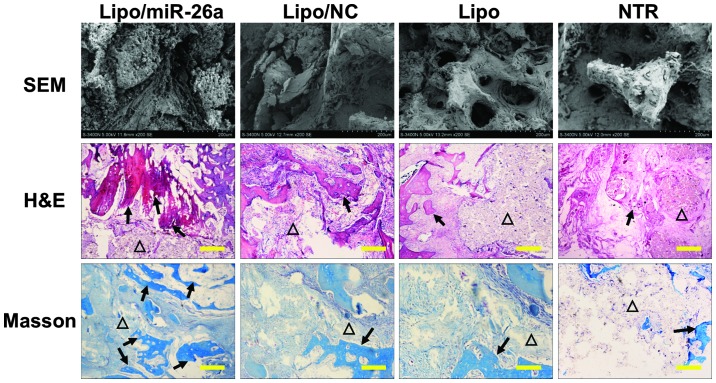
SEM observation of the residual tissues on the scaffold and histological evaluation of new bone formation (black arrow) within the scaffold (triangle) by H&E and Masson's trichrome staining. Scale bar=100 *µ*m. SEM, scanning electron microscopy; H&E, hematoxylin and eosin; NC, negative control; NTR, non-treated; miR, microRNA; Lipo, Lipofectamine 2000.

The scaffold was collected 12 weeks post implantation and the residual tissue on the scaffold was observed by SEM. The scaffold pores were filled with abundant ECM, particularly in the Lipo/miR-26a group in which the surface was covered with large collagen fibers and the pores were not visible (Fig. 4). The pores could still be detected in the Lipo/NC, Lipo and NTR group (Fig. 4). The *in vivo* bone anabolic ability was analyzed by H&E and Masson's trichrome staining to visualize new bone regeneration. All the groups healed well without apparent inflammatory granuloma (Fig. 4). In line with the *in vitro* observation, a larger area and notable new bone formation was observed in the Lipo/miR-26a-transfected group under H&E staining (black arrows) and Masson's trichrome staining (black arrows) compared with the control groups (Fig. 4).

## Discussion

The ideal effect of bone defect repair is to induce autologous bone regeneration instead of exogenous replacement. The development of stem cell technology has made it possible for multilineage MSCs to differentiate into osteoblasts and osteocytes to formulate the new bone tissue. However, bone regeneration often requires a large number of MSCs and despite MSCs being universally expressed in numerous tissues, in the majority of cases they are difficult to isolate. Although *in vitro* expansion can increase the cell number, long term proliferation and continuous passage have adverse effects on the normal function of BMSCs (19). By contrast, ASCs are derived from adipose tissue and can be easily obtained in a large number. Therefore, ASCs are considered to be an ideal alternative source of cells to BMSCs for tissue engineering.

In addition, to improve the function of ASCs in bone tissue engineering, the osteogenic capability of ASCs requires improvement. A previous study demonstrated that ASCs are transfected by a recombinant adenovirus vector containing human BMP2 (13). However, as the BMP2 gene is exogenous, this may potentially lead to genome modifications. miRNAs represent powerful endogenous therapeutic molecules that can regulate multiple target genes. It is hypothesized that their application *in vivo* may better mimic natural regulatory pathways as they are endogenous regulators in cells (20). In addition, the differentiation of stem cells may even be accelerated by miRNA treatment (21). Hence, miRNA is a promising molecule for improving the function of ASCs. In the present study, miR-26a, a novel osteogenic-angiogenic promoting molecule, was transfected into ASCs. The results demonstrated that the transfection of miR-26a markedly increased the osteogenic differentiation of ASCs *in vitro* without apparent effects on morphology or viability. The *in vivo* evaluation also demonstrated new bone formation following miR-26a transfection. Taken together, the results suggest that miR-26a can be applied to enhance the osteogenic capacity of ASCs.

Scaffolds are another critical element for bone regeneration. The ideal scaffold is able to support the defective area, allow the cells to penetrate, provide calcium and phosphorus elements and eventually degrade. Porous structures with an adequate pore diameter are beneficial for the penetration of cells and the circulation of nutrients (22). An accurate calcium phosphorus ratio is also important for new bone mineralization (23,24). HA, a frequently used scaffold for bone substitutes, is similar to the natural bone composition and has been developed numerous times (25–27). Consequently, the HA scaffold was selected in the present study to ensure that the results best demonstrate the capabilities of transfected ASCs.

In conclusion, the present study demonstrated that miR-26a can markedly increase the osteogenic differentiation ability of ASCs without apparent cytotoxicity *in vitro*. The combination of miR-26a-enhanced ASCs and an HA scaffold can significantly improve new bone formation, and thus may be used as a bone substitute for repairing bone defects.

## Figures and Tables

**Table I:** Primers used for reverse transcription-quantitative polymerase chain reaction.

Gene	Forward primer sequence (5′-3′)	Reverse primer sequence (5′-3′)
ALP	AACGTGGCCAAGAACATCATCA	TGTCCATCTCCAGCCGTGTC
COL1	GCCTCCCAGAACATCACCTA	GCAGGGACTTCTTGAGGTTG
OCN	GGTGCAGACCTAGCAGACACCA	AGGTAGCGCCGGAGTCTATTCA
BMP2	CAACACCGTGCTCAGCTTCC	TTCCCACTCATTTCTGAAAGTTCC
GAPDH	GGCACAGTCAAGGCTGAGAATG	ATGGTGGTGAAGACGCCAGTA

ALP, alkaline phosphatase; COL1, collagen I; OCN, osteocalcin; BMP2, bone morphogenetic protein 2; GAPDH, glyceraldehyde-3-phosphate dehydrogenase.
